# Effect of Parental Drinking on Adolescents

**Published:** 1996

**Authors:** Michael Windle

**Affiliations:** Michael Windle, Ph.D., is a senior research scientist at the Research Institute on Addictions, Buffalo, New York

**Keywords:** problematic AOD use, parent, adolescent, parenting skills, parent child relations, parental attitude, marital conflict, family dynamics, victim of abuse, AODR (alcohol and other drug related) family problems, role model, coping skills, protective factors, intervention

## Abstract

Adolescence brings with it many biological, psychological, and social changes. Parents continue to play an important role in their children’s development during this time. Parental problem drinking can adversely affect adolescent development and adjustment by interfering with parenting skills and marital relations. It also can lead parents to model ineffective coping strategies and other problem behaviors. Children with problem-drinking parents are at risk for alcohol and other drug use as well as for psychological problems. Protective factors, such as relatively stable patterns of family behavior around meals and holidays, can help offset the negative effects of parental drinking.

Many biological, psychological, and social changes characterize the phase in the life span known as adolescence. These changes include the onset of puberty, an increased self-identity, the initiation of dating, and the development of intimate relationships. Early theories of adolescent development described this period as one of “storm and stress” with regard to parent-child relations (see, for example, Douvan and Adelson 1966). More recent research has indicated that adolescents confront a host of challenging and sometimes unique events. Although they frequently prefer to handle these challenges on their own, adolescents often view parents as significant confidants and social support agents in times of crises (see Petersen 1988). Hence, although parents and adolescents may disagree over specific issues, such as curfew or amount of allowance, parents continue to play a salient role in the development of adolescents, just as they do with infants and young children. Problem drinking^1^ by parents, however, may disrupt this emerging pattern of parent-adolescent relations and adversely affect adolescent development and adjustment in several ways. The following sections describe how parents’ alcohol-related problems may influence adolescent development. Although this brief article focuses on parental influences on children, a more comprehensive (and longer) article would include the reciprocal influences of children on parents as well as dynamic parent-child relations across time (see, for example, Windle and Tubman in press).

## Parenting Skills

Problem drinking by parents may negatively influence important parenting skills that serve to nurture and provide guidance for children. For example, problem drinking may contribute to inconsistency or unpredictability in parenting behaviors (see Holmes and Robins 1987). On some occasions, an adolescent’s request to use the car may be met with verbal abuse by a parent; other times, the request may receive consideration and support. Under the influence of alcohol, some parents may become more (or less) tolerant of their child’s failure to perform household tasks or permissive with regard to their child’s consumption of alcoholic beverages. A range of research in related fields (e.g., on the relationship between maternal depression and child functioning) indicates that such inconsistency in parenting may undermine a child’s sense of order, control, and stability in the family environment, reducing both feelings of self-esteem and perceptions of self-competence (for review, see Downey and Coyne 1990).

In addition to inconsistent and unpredictable parenting behaviors, parental alcohol abuse may contribute to poorer monitoring of adolescent behaviors. Parental monitoring includes establishing rules for appropriate and inappropriate behaviors for adolescents (e.g., drinking practices, curfew, and household responsibilities), consistently enforcing established penalties for violating the rules, and overseeing friendship and peer-group choices and leisure activities. The research literature has consistently indicated that higher levels of parental monitoring are associated with lower levels of adolescent alcohol and other drug use as well as other forms of delinquent behavior (see Dishion and Loeber 1985). Furthermore, research has shown that higher levels of parental monitoring increase the likelihood that children will select fewer peers who use alcohol or other illicit substances (see Dishion and Loeber 1985; Kandel and Andrews 1987). Parental monitoring helps establish an orderly structure whereby adolescents are more easily able to distinguish healthy from unhealthy choices pertaining to drinking, peer groups, and other forms of risky behavior (e.g., sexual activity). By decreasing monitoring activities, parental problem drinking may undermine healthy adolescent adjustment.

Problem-drinking parents also may provide lower levels of parental nurturing and emotional availability, thereby increasing the risk for adolescent drinking. As mentioned previously, adolescents continue to rely on parents for emotional support to help with challenges, such as handling conflict with others, and for guidance in important future-oriented decisions. Higher levels of parental nurturance and warmth of expression consistently have been associated with lower levels of alcohol and substance use and higher levels of general mental health and well-being among adolescents (see Barnes 1990; Brook et al. 1990). Parents who abuse alcohol typically provide less nurturance to their offspring. They are more often “emotionally unavailable” as a result of drinking-related consequences, which include hangovers, irritability, and negative mood states. These effects disrupt healthy emotional development in their children. To compensate for the lack of parental affection and support, adolescents may tend to affiliate with friends who drink more heavily. The child is thus propelled on a path of continual adverse outcomes.

Along with a lack of nurturance, parental use of harsh discipline also has been associated with patterns of heavier parental alcohol use. Parents who are inebriated may exercise poorer judgment in meting out punishment to children and may become less inhibited and overly aggressive. Harsh disciplinary practices are linked to a range of adverse outcomes among children, including a greater emotional distance between parents and offspring, higher levels of aggressive behavior by children, poorer school performance, and early affiliation with deviant peer groups (see Patterson 1986). These effects also may foster the early onset and rapid acceleration of adolescent alcohol abuse (see Holmes and Robins 1987).

Problem-drinking parents frequently demonstrate a greater tolerance of adolescent drinking and other substance use. In this way, they provide implicit approval for their children’s alcohol use. Research has consistently found that higher levels of parental tolerance of adolescent drinking are associated with an earlier onset of drinking among offspring as well as with the escalation to higher levels of alcohol use.^2^ Increased alcohol use, in turn, can lead to more alcohol-related adverse social consequences, such as problems at school or with legal authorities (see Barnes and Welte 1986; Johnson and Pandina 1991).

## Marital and Family Functioning

In addition to affecting parenting skills adversely, problem drinking by parents may negatively affect child and adolescent development by impairing marital and family relations. For example, higher levels of alcohol use by parents have been associated with higher levels of marital conflict (see Leonard 1993; Reich et al. 1988). In turn, higher levels of marital conflict have been associated with higher levels of child and adolescent alcohol use and aggression. Marital conflict has a threatening and destabilizing influence on families. The fears of children in these families illustrate this effect; frequently, such children express concern both for the breakup of the family unit and for their personal safety. Prolonged marital conflict, influenced by parental alcohol use, may contribute to children’s attempts to escape these aversive conditions through personal alcohol use with peers, who may be perceived as more emotionally supportive.

Heightened levels of marital conflict also may contribute to spousal or child physical abuse, thereby creating other risky family conditions for child and adolescent alcohol abuse. Research has fairly consistently indicated a high rate of alcohol use in families characterized by spousal and child abuse (for review, see Widom 1993). Although numerous explanations have been suggested for these findings (see Miller 1993; Widom 1993), there is little question either of the consistency of the association or of the adverse consequences for offspring in a family, regardless of which family members are the victims of abuse. Heavy alcohol use is an all-too-common factor in the intergenerational transmission of violence, such that alcohol-and-violence begets alcohol-and-violence.

The persistent heavy use of alcohol by parents may undermine healthy family relations by producing financial strains. Job loss associated with alcohol-abusing behaviors directly affects family revenue; without intervening treatment, a parent may have difficulty obtaining a new job. Such treatment and associated loss of wages may be costly—both financially and emotionally—to family members. Even if alcohol-abusing behavior does not result in job loss, family financial strains may occur through uncompensated “missed” days of work, alcohol-related medical costs, and the costs associated with purchasing large quantities of alcohol. Financial stress also may contribute to an increased level of marital conflict. Thus, parental alcohol abuse negatively affects both family and work contexts and may thereby indirectly influence the financial and overall emotional stability of the home environment.

Parents may contribute to adolescent drinking even before the child is born by selecting a problem-drinking partner (McKenna and Pickens 1983). This concept, described as “nonrandom partner selection” (i.e., assortative mating), refers to research findings indicating that alcoholics and problem drinkers are more likely to marry partners who abuse alcohol (see Hall et al. 1983). Assortative mating may increase the likelihood of adverse outcomes among offspring by increasing both genetic and environmental risk. Genetic risk is increased because the offspring may inherit a genetic predisposition toward alcoholism through the combined lineages of the maternal and the paternal sides of the family. Environmental risk is increased in that the rearing environment of children raised by two alcoholic or problem-drinking parents may be severely compromised with regard to parenting skills, yielding a “double jeopardy” situation for the development of the offspring. In addition, if both parents have drinking problems, then the potential stress-buffering or moderating influences of a nondrinking parent are not present in the family.

Ultimately, the disruptive effects of problem drinking on marital relations and family functioning may influence adolescents’ perceptions of how families typically function. Some adolescents may come to view the marital and family dysfunction they experience as normative. This experience then becomes a “blueprint” for their own intimate relationships and behavior with regard to major events such as marriage and parenthood. An adolescent who observes or experiences higher levels of parental hostility, verbal abuse, or physical abuse may therefore adopt such an orientation and set of expectations with his or her spouse and children; in this way, beliefs and expectations about parental alcohol use and family interactions may be transmitted from generation to generation.

## Role Modeling

Problem-drinking parents may affect adolescent drinking through basic socialization and learning processes as well as through role modeling. Consistent positive associations between parental and adolescent levels of alcohol use have been reported in the research literature (see Kandel 1980). Some researchers have suggested that many adolescents are simply imitating their parents’ behavior because they see their parents as powerful figures to emulate (see Hansen et al. 1987). Other research has found that higher levels of parental alcohol use are associated with the earlier acquisition and elaboration of knowledge about alcohol use by children as young as preschool age. For instance, research has shown that preschool children with parents who drink alcohol are better able to identify alcoholic beverages (using a “blinded” smell test) than are preschoolers with parents who do not consume alcohol (Noll et al. 1990). Exposure to parental alcohol use also is associated with children’s intentions to drink alcohol and their perception of alcohol consumption as a positive activity (see Gaines et al. 1988).

Parents who drink alcohol to cope with stressful life events also may affect their children through modeling an ineffective coping strategy. That is, children who observe self-medicating with alcohol in response to stress may perceive that such behavior is an effective way to confront distressing circumstances; they thus may be more likely to adopt the coping mechanism themselves. The difficulties with such a coping strategy are twofold. First, self-medicating with alcohol may provide short-term relief from “pressures,” or stressors, but is not likely to be useful in generating constructive solutions that may ameliorate or eliminate the distress or its source. Second, adolescents’ reliance on alcohol to self-medicate for depressed moods may contribute to more frequent and serious alcohol use and associated problems. These problems may, in turn, contribute to escalating levels of stressful life events (e.g., poorer school performance or contact with legal authorities) that may foster increased self-destructive use of alcohol (Khantzian 1985).

## Protective Factors

Although much of the research literature has identified factors associated with parental problem drinking that negatively affect outcomes for children and adolescents, some research has attempted to identify family (and non-family) factors that “protect” high-risk children, such as children of alcoholics, from adverse outcomes. For example, Wolin and colleagues (1979) reported that families with alcoholic members who retained relatively stable patterns of behavior around everyday activities, such as meals, and the celebration of special events, such as births, marriages, and holidays (i.e., family rituals) had offspring with higher levels of adjustment and fewer alcohol problems than did families whose family rituals were disrupted by the alcoholic family member. Hence, the preservation of family rituals provided more stability, predictability, and perceived support for these offspring.

Other family related protective factors include the strength of positive emotional ties (e.g., warmth and nurturance) with family members, consistent and fair rules for adolescent conduct, and “open” parent-child communication styles (see Barnes 1990). The emotional strength of the parent-child relationship has been related to delays in the age of onset of substance use and to a decreased probability of escalation to more serious levels of use (see Brook et al. 1990). Similarly, parental monitoring of, and clear and fair guidelines for, adolescent behaviors (e.g., with regard to curfew or household tasks) have been associated with lower levels of alcohol and other substance use and with a less deviant peer network. Parent-child communication plays a significant role both in establishing and maintaining positive emotional ties and in promoting mutual understanding of rules of conduct. Adolescents are more likely to communicate openly with parents if they believe that they will not be criticized and belittled. Likewise, open discussion regarding adolescent conduct (e.g., curfew) may facilitate understanding (if not agreement) by adolescents and perhaps a greater sense of involvement and participation in decisions that directly affect their lives.

## Summary

Adolescent behaviors, including alcohol use and abuse, are influenced by a multitude of biological, psychological, and sociocultural factors. Furthermore, not all adolescents are influenced by the same set of factors. For some problem-drinking adolescents, parental role-modeling behaviors may be more influential, whereas for others, disrupted family relations (e.g., marital conflict) may have more influence. In addition, current knowledge is limited with regard to how adolescent drinking behavior is related to adult alcohol abuse or other manifestations of maladjustment (e.g., depression or criminality). Nevertheless, it is evident that parental alcohol abuse may have a range of potential adverse effects on adolescents. Problem drinking by parents may influence role-modeling behaviors, parenting skills, and marital and family relations, all of which may contribute to a host of problematic outcomes for adolescents. Without appropriate parent-child or family based interventions, these disruptive, alcohol-influenced parenting behaviors may contribute to internalizing problems (e.g., depression and anxiety disorders) and externalizing problems (e.g., delinquency) among children and adolescents, including early onset of alcohol use and a rapid acceleration to problematic use throughout adolescence and into adulthood.

## Footnotes

1 For the purposes of this article, the terms “problem drinking,” “alcohol abuse,” and “heavy alcohol use” are used interchangeably to refer to drinking behavior associated with adverse social consequences, dependency symptoms, or both.

2 For adolescents, the term “higher levels of alcohol use” refers to binge drinking and other drinking behavior associated with adverse social consequences.

## References

[b1-arhw-20-3-181] Barnes GM, Collins RL, Leonard KE, Searles JS (1990). Impact of the family on adolescent drinking patterns. Alcohol and the Family: Research and Clinical Perspectives.

[b2-arhw-20-3-181] Barnes GM, Welte JW (1986). Patterns and predictors of alcohol use among 7–12th grade students in New York State. Journal of Studies on Alcohol.

[b3-arhw-20-3-181] Brook J, Brook D, Gorton A, Whiteman M, Cohen P (1990). The psychosocial etiology of adolescent drug use: A family interactional approach. Genetic, Social, and General Psychology Monographs.

[b4-arhw-20-3-181] Dishion TJ, Loeber R (1985). Adolescent marijuana and alcohol use: The role of parents and peers revisited. American Journal of Drug and Alcohol Abuse.

[b5-arhw-20-3-181] Douvan E, Adelson J (1966). The Adolescent Experience.

[b6-arhw-20-3-181] Downey G, Coyne JC (1990). Children of depressed parents: An integrative review. Psychological Bulletin.

[b7-arhw-20-3-181] Gaines LS, Brooks PH, Maisto S, Dietrich M, Shagena M (1988). The development of children’s knowledge of alcohol and the role of drinking. Journal of Applied Developmental Psychology.

[b8-arhw-20-3-181] Hall RL, Hesselbrock VM, Stabenau JR (1983). Familial distribution of alcohol use: I. Assortative mating in the parents of alcoholics. Behavior Genetics.

[b9-arhw-20-3-181] Hansen WB, Graham JW, Sobel JL, Shelton DR, Flay BR, Johnson CA (1987). The consistency of peer and parent influences on tobacco, alcohol, and marijuana use among young adolescents. Journal of Behavioral Medicine.

[b10-arhw-20-3-181] Holmes JS, Robins LN (1987). The influence of childhood disciplinary experience on the development of alcoholism and depression. Journal of Child Psychology and Psychiatry.

[b11-arhw-20-3-181] Johnson V, Pandina RJ (1991). Familial and personal drinking histories and measures of competence in youth. Addictive Behaviors.

[b12-arhw-20-3-181] Kandel DB (1980). Drug and drinking behavior among youth. Annual Review of Sociology.

[b13-arhw-20-3-181] Kandel DB, Andrews K (1987). Processes of adolescent socialization by parents and peers. International Journal of Addictions.

[b14-arhw-20-3-181] Khantzian EJ (1985). The self-medication hypothesis of addictive disorders: Focus on heroin and cocaine dependence. American Journal of Psychiatry.

[b15-arhw-20-3-181] Leonard KE, Martin SE (1993). Drinking patterns and intoxication in marital violence: Review, critique, and future directions for research. Alcohol and Interpersonal Violence: Fostering Multidisciplinary Perspectives.

[b16-arhw-20-3-181] McKenna T, Pickens R (1983). Personality characteristics of alcoholic children of alcoholics. Journal of Studies on Alcohol.

[b17-arhw-20-3-181] Miller B, Martin SE (1993). Investigating links between childhood victimization and alcohol problems. Alcohol and Interpersonal Violence: Fostering Multidisciplinary Perspectives.

[b18-arhw-20-3-181] Noll RB, Zucker RA, Greenberg GS (1990). Identification of alcohol by smell among preschoolers: Evidence for early socialization about drugs occurring in the home. Child Development.

[b19-arhw-20-3-181] Patterson GR (1986). Performance models for antisocial boys. American Psychologist.

[b20-arhw-20-3-181] Petersen A (1988). Adolescent development. Annual Review of Psychology.

[b21-arhw-20-3-181] Reich W, Earls F, Powell J (1988). A comparison of the home and social environments of children of alcoholic and nonalcoholic parents. British Journal of Addiction.

[b22-arhw-20-3-181] Widom CS, Martin SE (1993). Child abuse and alcohol use and abuse. Alcohol and Interpersonal Violence: Fostering Multidisciplinary Perspectives.

[b23-arhw-20-3-181] Windle M, Tubman JG, Silverman WK, Ollendick T Children of alcoholics. Developmental Issues in the Clinical Treatment of Children and Adolescents.

[b24-arhw-20-3-181] Wolin SJ, Bennett LA, Noonan DL (1979). Family rituals and the recurrence of alcoholism over generations. American Journal of Psychiatry.

